# The Long Noncoding RNA MEG3 Retains Epithelial-Mesenchymal Transition by Sponging miR-146b-5p to Regulate SLFN5 Expression in Breast Cancer Cells

**DOI:** 10.1155/2022/1824166

**Published:** 2022-08-18

**Authors:** Xuefeng Gu, Jingyi Li, Xiaojia Zuo, Kaijie Chen, Guoqing Wan, Li-li Deng, Weiming Zhao, Changlian Lu

**Affiliations:** ^1^Shanghai Key Laboratory of Molecular Imaging, Zhoupu Hospital, Shanghai University of Medicine and Health Sciences, China; ^2^Qiqihar Medical University, China; ^3^School of Clinical Medicine, Guizhou Medical University, China; ^4^Department of Oncology, The Second Affiliated Hospital of Harbin Medical University, China; ^5^Heilongjiang University of Traditional Chinese Medicine, China

## Abstract

More and more studies have shown that long noncoding RNAs (lncRNAs) play essential roles in malignant tumors. The lncRNA MEG3 serves as a crucial molecule in breast cancer development, but the specific molecular mechanism needs to be further explored. We previously reported that Schlafen family member 5 (SLFN5) inhibits breast cancer malignant development by regulating epithelial-mesenchymal transition (EMT), invasion, and proliferation/apoptosis. Herein, we demonstrated that MEG3 was downregulated in pan-cancers and correlated with SLFN5 expression positively in breast cancer by bioinformatics analysis of TCGA and UCSC Xena data. Intervention with MEG3 positively affected SLFN5 expression in breast cancer cells. MEG3 repressed EMT and migration/invasion, similar to our previously reported functions of SLFN5 in breast cancer. Through bioinformatics analysis of starBase and LncBase data, 12 miRNAs were found to regulate both SLFN5 and MEG3, in which miR-146b-5p was confirmed to be regulated by MEG3 using MEG3 siRNA and overexpression method. MiR-146b-5p could bind to both SLFN5 3′UTR and MEG3, and inhibit their expression in a competing endogenous RNA mechanism, assayed by luciferase reporter and RNA pull down methods. Therefore, we conclude that MEG3 positively modulates SLFN5 expression by sponging miR-146b-5p and inhibits breast cancer development.

## 1. Introduction

Although breast cancer (BRCA-)-related mortality has been declining over the past 20 years, BRCA has the highest morbidity and mortality in woman cancers worldwide to date [1, 2]. Treatment failure is mainly due to the extensive heterogeneity and distant metastases of BRCA [3]. For cancer cell metastasis, epithelial-mesenchymal transition (EMT) is the initial step, through which cancer cells acquire mesenchymal morphologies, migratory and invasive capabilities, and can metastasize to other distal organs [4–7]. In BRCA, EMT triggers primitive alterations in the tumor microenvironment, which increases the number of tumor cells and their migration, invasion potential, and chemoresistance [8, 9].

Studies show that the expression or dysfunction of long noncoding RNAs (lncRNAs) is correlated to many serious diseases, such as degenerative neurological diseases, cardiovascular diseases, and cancer [10–13]. Various lncRNAs play roles in regulating EMT and tumor progression in a variety of tumors, but the mechanisms are different [14]. For example, the lncRNA SNHG7 promotes BRCA tumorigenesis and progression through the Notch-1 signaling pathway and EMT initiation [15]. The lncRNA PANDAR promotes the EMT pathway by upregulating MMPs (2 and 9) levels in BRCA [16]. Maternally expressed gene 3 (MEG3) affects cell growth and development in various tissues [17, 18]. MEG3 levels are downregulated in a variety of cancers [19–21]. In BRCA, MEG3 inhibits BRCA growth by upregulating endoplasmic reticulum stress and activating p53 [22] and suppresses EMT of BRCA cells by targeting E-cadherin [23]. However, the mechanism through which MEG3 regulates BRCA progression remains to be explored.

Schlafen-5 (SLFN5), a member of the Schlafen family, is abnormally expressed and involved in the progression of melanoma, renal cell carcinoma, gastric cancer, and glioblastoma [24–27]. BRCA big data from the Cancer Genome Atlas (TCGA) shows that SLFN5 transcript level significantly decreases in BRCA. We previously found SLFN5 inhibited MT1-MMP expression associated invasion [28], and retained ZEB1 transcriptional expression associated EMT and progression in BRCA cells [29, 30], so SLFN5 may play an inhibitory role in BRCA progression. However, the upstream regulatory mechanism of SLFN5 is rarely explored, and whether SLFN5 is regulated by noncoding RNA, such as lncRNAs and microRNA, in BRCA is unclear.

Herein, by means of bioinformatics analysis, we found that MEG3 and SLFN5 present the same expression pattern in BRCA and bind with the common mircoRNA, laying a foundation for the competitive endogenous RNA mechanism. Our hypothesis was proved by a series of biological experiments. MEG3 intervention can affect the SLFN5 level in BRCA cells and regulate the EMT process. Importantly, miR-146b-5p can bind both MEG3 and SLFN5 and regulate their expression via a competitive mechanism, suggesting a novel regulation mechanism for SLFN5 in BRCA.

## 2. Materials and Methods

### 2.1. Bioinformatics Assay

All original data were downloaded from TCGA (https://cancergenome.nih.gov/) and UCSC Xena (http://xena.ucsc.edu/) websites and integrated using R 4.1.0 to verify the results presented in the website for the database. The clinical characteristics associated with MEG3 expression in BRCA patients are listed in Table 1. We input MEG3 into the “Quick PanCAN Analysis” module of the UCSCXenaShiny and obtained the differences of MEG3 in tumors compared with adjacent normal tissues. Using the “Expression Analysis-Box Plot” module of GEPIA2 Web server (http://gepia2.cancer-pku.cn/#analysis), the box plot of BRCA tumor and normal tissue expression difference was obtained. The relationship between SLFN5 and MEG3 in BRCA was obtained by calculating the Pearson correlation coefficient. ESTIMATE, an algorithm designed for predicting TME tumor purity, provided both immune and stroma scores for this study.

### 2.2. Cell Culture and Transfection

Four types of human BRCA cells (MDA-MB-231, BT-549, MCF-7, and T-47D) and 293T cells were all from ATCC and cultured in Dulbecco's modified Eagle's medium (Gibco: C11885500BT, Australia) supplemented with 10% fetal bovine serum (Gibco: 10099-141, Australia). SiRNAs targeting MEG3 and SLFN5, miR-146b-5p mimic and inhibitor were synthesized (GenePharma, China), sequences as shown in Table 2. Transfection experiments were performed using Lipofectamine™ 2000 (Invitrogen, USA) when cell confluence reaches 30% in six-well plates.

### 2.3. Real-Time PCR

2 × 10^6^ cells were lysed with 1 ml TRIzol reagent (Invitrogen) for total RNA isolation. RNA was reversely transcribed into cDNA using the PrimeScript RT reagent kit (TaKaRa, Japan), and expression difference of RNA was quantitatively analyzed by real-time PCR using a SYBR PremixEx Taq kit (Takara, Japan). Primer sequences used are shown in Table 3. Relative fold change of RNA was calculated using formula 2^−*ΔΔ*Ct^ with *β*-actin or U6 as loading control.

### 2.4. Western Blotting

Cells were lysed using RIPA lysis buffer (Beyotime, China), and proteins were extracted. Proteins were isolated by SDS-PAGE and transferred to PVDF membranes (Beyotime, China). After blocking with 5% nonfat milk, PVDF membranes were incubated with SLFN5 rabbit pAb (Sigma-Aldrich: HPA017760), E-cadherin rabbit mAb (CST: 3195), vimentin rabbit mAb (CST: 5741), ZEB1 rabbit mAb (CST: 3396), and *β*-actin rabbit mAb (CST: 4970S) at 4°C for above 12 hours. After incubation with HRP-conjugated anti-rabbit antibody at room temperature for 1 h, the membranes were then incubated with enhanced chemiluminescent (ECL, Pierce, USA) for protein band detection.

### 2.5. RNA Pull down Assay

MEG3 sequences were amplified and ligated into pcDNA3.1 vector, and recombinant vectors were transformed into JM109 competent cell. MEG3 plasmids and empty plasmids (NC) were purified, and plasmids were linearized with the restriction endonuclease *Sma*I. Linearized plasmids were used as templates for MEG3 RNA and NC transcriptions, respectively, using T7 RNA polymerase (Beyotime: D7069, China). Both MEG3 RNA and NC RNA were labeled with biotin at 3′ end using desthiobiotinylation kit (Pierce: 20163). The biotin-labeled RNAs were combined with streptavidin magnetic beads to pull down RNA isolated from MCF7 cells. Pulled down RNAs were isolated from magnetic beads with proteinase K treatment and reverse transcribed into cDNA used for the following real-time PCR to detect miR-146b-5p expression using SYBR Green (ABI: 4387406).

### 2.6. Dual-Luciferase Reporter Assay

Both the sequence of SLFN5 3′UTR and MEG3, putative binding sites for miR-146b-5p, were cloned into the pmirGLO Dual-Luciferase miRNA Target Expression vector (Promega: E1330, Wisconsin, USA) to obtain the MEG3-WT and SLFN5-WT constructs, respectively. The mutant (MUT) sequences of MEG3 and SLFN5 3′UTR were introduced to create the MEG3-MUT and SLFN5-MUT constructs, respectively. These constructs were transfected into 293T cells with miR-146b-5p mimics using Lipofectamine™ 2000 (Invitrogen: 11668-019). The luciferase activity was assayed by Dual-Luciferase® Reporter System (Promega: E1910) using GloMaxTM (Promega, E5331).

### 2.7. Migration and Invasion Assays

For migration assay, 5 × 10^4^ cells were placed in a 24-well transwell plate (Corning Costar: 3422) and incubated for 24 hours. For invasion assay, 80 *μ*l of cold gel matrix (BD: 35623) was transferred to the transwell insert. After gelling, 5 × 10^4^ cells were inoculated on the gel matrix and cultured for 48 hours. Following incubation for above indicated time, the cells on the upper surface of filter were removed by cotton swabs, and the filter was stained with 0.4% crystal violet. Migrated or invaded cells on the lower surface were photographed with a Leica light microscope, and cell numbers were counted.

### 2.8. Statistical Analysis

Data are presented as the mean ± SD of three independent experiments and analyzed using R (version 4.1.0) and SPSS (version 17.0) software with *P* < 0.05 as statistically significant difference. The correlation between two groups was analyzed by Pearson *χ*2 test. Two groups' comparison was analyzed by *t* test. Multiple groups' comparison was performed by one-way ANOVA combined with Tukey's multiple comparisons test.

## 3. Results

### 3.1. MEG3 Is Downregulated in Pan-Cancers and Positively Correlated with SLFN5 Expression in BRCA

**Figure 1:**
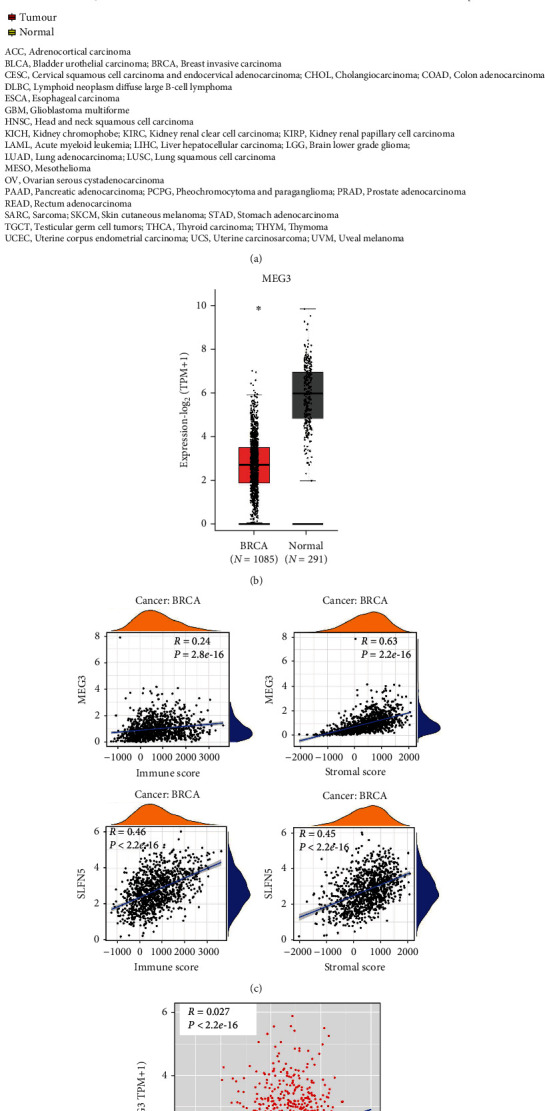
The MEG3 expressed in pan-cancers and the correlation with SLFN5 in BRCA based on TCGA database and UCSC Xena website. (a) Analysis of MEG3 expression in 33 types of tumor tissues compared with normal tissues, decreased in 24 types of tumor tissues (such as BRCA, LUAD, LUSC, and OV), increased in 4 types of tumor tissues (CHOL, DLBC, TGCT, and THYM), with no significant difference in 3 types of tumors (HNSC, PCPG, and SARC), and no normal group information in the remaining 2 types of tumors (MESO and UVM). (b) Analysis of MEG3 expression in 1085 BRCA and 291 normal tissues. (c, d) The correlation analysis between TME (immune score and stromal score) and MEG3 or SLFN5 in BRCA. ∗*P* < 0.05, ∗∗*P* < 0.01, ∗∗∗*P* < 0.0001. Abbr. TCGA: The Cancer Genome Atlas; TME: tumor microenvironment. BRCA: breast cancer.

Firstly, we analyzed the difference of MEG3 expression between 33 tumor tissues and normal tissues, showing a decrease in 24 types of tumor tissues (such as BRCA, lung adenocarcinoma (LUAD), lung squamous cell carcinoma (LUSC), and ovarian cancer (OV)) and an increase in 4 types of tumor tissues (cholangio carcinoma (CHOL), large B-cell lymphoma (DLBC), testicular cancer (TGCT), and thymoma (THYM)), with no significant difference in 3 types of tumors (head and neck cancer (HNSC), pheochromocytoma and paraganglioma (PCPG) and sarcoma (SARC)) and no normal group information in the remaining 2 types of tumors (mesothelioma (MESO) and ocular melanomas (UVM)). These indicate that MEG3 may be negatively correlated with most tumor progression (Figure 1(a)). Subsequently, the expression level of MEG3 was further confirmed to be significantly lower in BRCA patients by bioinformatics (Figure 1(b)). It must also be mentioned that MEG3 level was negatively associated with TNM stage (Table 1 and Figure S1). Moreover, both MEG3 and SLFN5 present similar positive correlationships with immune infiltration in the TME of BRCA based on immune score and stromal score (Figure 1(c)). It should be pointed out that our previous study reported that SLFN5 level was negatively correlated with tumor stage in BRCA [29]. The results of Pearson analysis revealed that the MEG3 transcript was correlated with SLFN5 mRNA level positively in BRCA (Figure 1(d)), indicating that MEG3 may be involved in progression and positively associated with SLFN5 regulation in BRCA.

### 3.2. Intervention with MEG3 Positively Affects SLFN5 Expression in BRCA *In Vitro*

**Figure 2:**
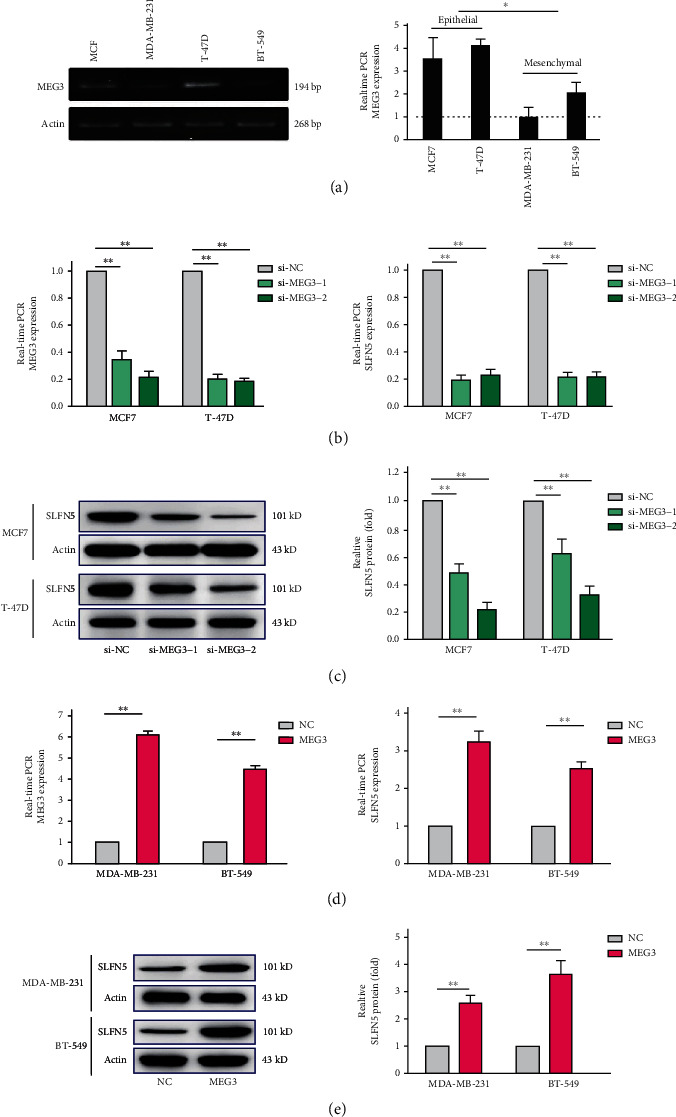
Intervention with MEG3 affects SLFN5 expression in BRCA cells. (a) Real-time PCR analysis of MEG3 RNA level in BRCA cell lines with different invasive capabilities, high-invasive capability cell lines MDA-MB-231 and BT549, and low-invasive capability cell lines MCF7 and T-47D. (b) Expression change of MEG3 RNA and SLFN5 mRNA in T-47D cells and MCF7 cells interfered with si-MEG3 or negative control siRNA (si-NC) analyzed by real-time PCR. (c) SLFN5 protein change in T-47D cells and MCF7 cells interfered with si-MEG3 or si-NC analyzed by Western blotting. (d) Expression change of MEG3 RNA and SLFN5 mRNA in BT-549 cells and MDA-MB-231 cells treated with MEG3 plasmids or control plasmids (NC) analyzed by real-time PCR. (e) SLFN5 protein change in BT-549 cells and MDA-MB-231 cells treated with MEG3 or NC plasmids analyzed by Western blotting. ∗∗*P* < 0.01.

The above data demonstrated that MEG3 is positively correlated with SLFN5 expression in BRCA, and to investigate the potential mechanism, MEG3 levels were detected in BRCA cell lines with different invasive capabilities. The results showed that MEG3 RNA levels were evidently lower in high-invasive BRCA cells (BT549 and MDA-MB-231) than that in low-invasive BRCA cells (T-47D and MCF7) (Figure 2(a)). Then, to research the function of MEG3 in BRCA cells, low-invasive BRCA cells were transfected with si-MEG3 plasmid, si-MEG3-1, and si-MEG3-2; meanwhile, high-invasive cells were transfected with MEG3 plasmid. Interestingly, in the lower invasive cells, the mRNA and protein levels of SLFN5 were both significantly downregulated after si-MEG3 transfection (Figures 2(b)–2(c)), si-MEG3-2 showing a higher transfection efficiency so chosen to be used in the following experiments. In contrast, MEG3 transfection led to SLFN5 mRNA and protein expression evidently upregulated in high-invasive BRCA cells (Figures 2(d)–2(e)), suggesting that MEG3 level positively regulates SLFN5 expression and possibly involved in invasion capability in BRCA *in vitro*.

### 3.3. MEG3 Represses BRCA Cell EMT and Invasion Similar to SLFN5 Functions in BRCA Cells

**Figure 3:**
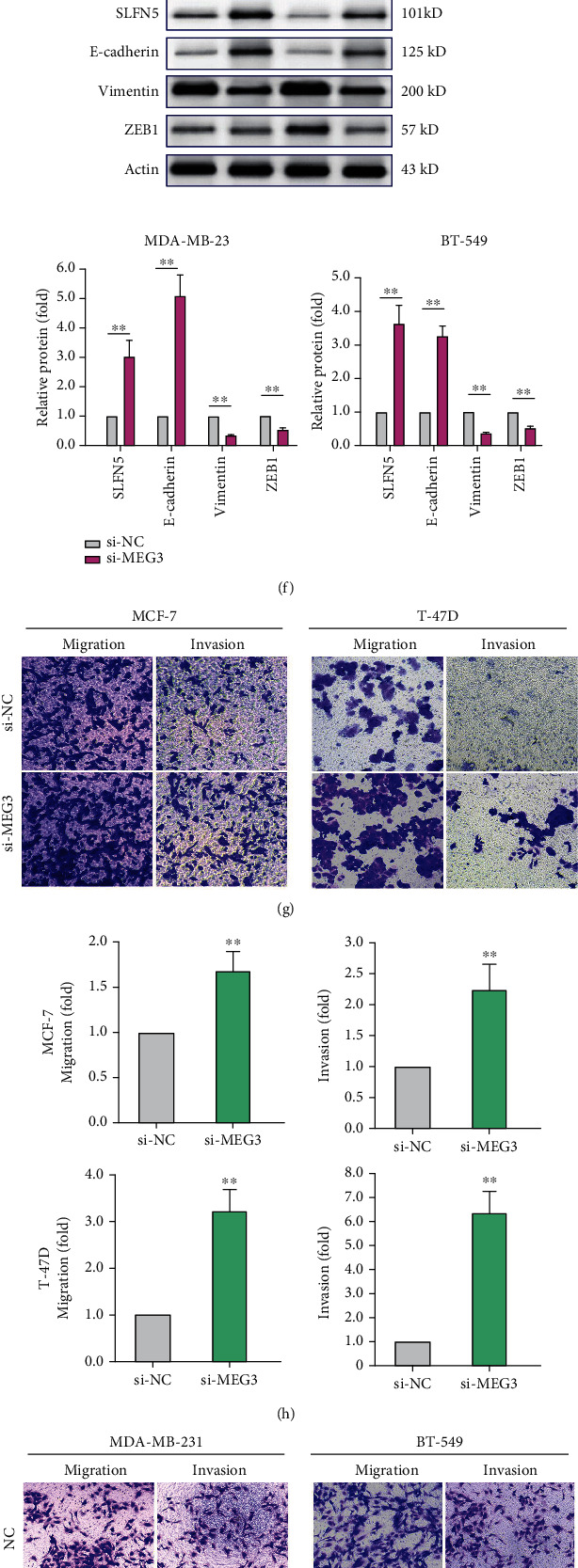
MEG3 represses BRCA cell migration, invasion, and EMT. (a, b) Morphological changes responding to si-MEG3 or si-NC interference (a) and MEG3 plasmid transfection (b) in indicated BRCA cells. (c, d) Relative mRNA level change of EMT molecular markers, vimentin, E-cadherin, and ZEB1, responding to si-MEG3 interference (c) or MEG3 plasmid transfection (d) in indicated BRCA cells. (e, f) Western blotting analyses of protein change of EMT markers responding to si-MEG3 transfection (e) or MEG3 plasmid transfection (f). (g–j) Cell migration and invasion capabilities evaluated by transwell assays responding to si-MEG3 (g, h) or MEG3 plasmid transfection (i, j). (k) Morphological changes of MCF-7 and T-47D cells responding to si-SLFN5 or si-NC interference. (l, m) Real-time and Western blotting analyses of vimentin, E-cadherin, and ZEB1, responding to si-SLFN5 or si-NC interference. ∗*P* < 0.05, ∗∗*P* < 0.01.

The above experiment results confirmed that the RNA expression of MEG3 in BRCA cells with low-invasive capability was significantly different from that in BRCA cells with high-invasive capability, which suggests that MEG3 may be associated with EMT process in BRCA. To verify this hypothesis, low-invasive cells with high MEG3 expression were knocked down by si-MEG3-2 or si-NC, and high-invasive cells were transfected with MEG3 plasmid or NC to observe the morphological changes and EMT-related gene expressions. The morphologies of si-MEG3-transfected cells changed from epithelial morphology to a dispersed and prolonged cell phenotype with mesenchymal characteristics (Figure 3(a)). In contrast, MEG3-transfected cells were partially transformed from long fusiform or spindle-shaped to regular paving stone morphology (Figure 3(b)). As morphology and EMT molecular markers, E-cadherin for epithelial and vimentin and ZEB1 for mesenchymal were examined and exhibited corresponding changes in mRNA levels (Figures 3(c) and 3(d)) and protein levels (Figures 3(e)–3(f)) after si-MEG3 or MEG3 treatment. Further, transwell migration and invasion assays were performed. Compared with NC group, si-MEG3-transfected cells exhibited evidently elevated migration/invasion abilities (Figures 3(g) and 3(h)). However, migration and invasion were weakened after MEG3 upregulation (Figures 3(i) and 3(j)). These confirmed that MEG3 level could influence EMT program and invasion capability in BRCA *in vitro*.

Our previous study revealed that high or low expression levels of SLFN5 lead to epithelial or interstitial morphology in some cancer cells, which indicates that SLFN5 may exert an important function on cellular EMT and invasion [28, 29]. Since the above results show that MEG3 can influence SLFN5 expression both at mRNA and protein levels and influence BRCA cell EMT and invasion, so we need to confirm SLFN5 function on EMT to explore their relationship further. Herein, T-47D and MCF7cells were transiently transfected with SLFN5-specific siRNA (si-SLFN5) or negative control siRNA (si-NC). Si-SLFN5-treated cells displayed a mesenchymal phenotype compared to si-NC-treated cells (Figure 3(k) in Figure 3 continued). Si-SLFN5 cells exhibited a decrease in vimentin and ZEB1 and an increase in E-cadherin at both mRNA and protein expression (Figures 3(l) and 3(m) in Figure 3 continued), confirming that SLFN5 knockdown can inhibit EMT in BRCA. These results showed that MEG3 and SLFN5 have similar functions on EMT in BRCA and, together with MEG3, can positively regulate SLFN5 expression, providing a full possibility for the regulatory mechanism of competitive endogenous RNAs (ceRNA).

### 3.4. MEG3 sponges miR-146b-5p leading to SLFN5 upregulation at the posttranscriptional level by ceRNA mechanism

**Figure 4:**
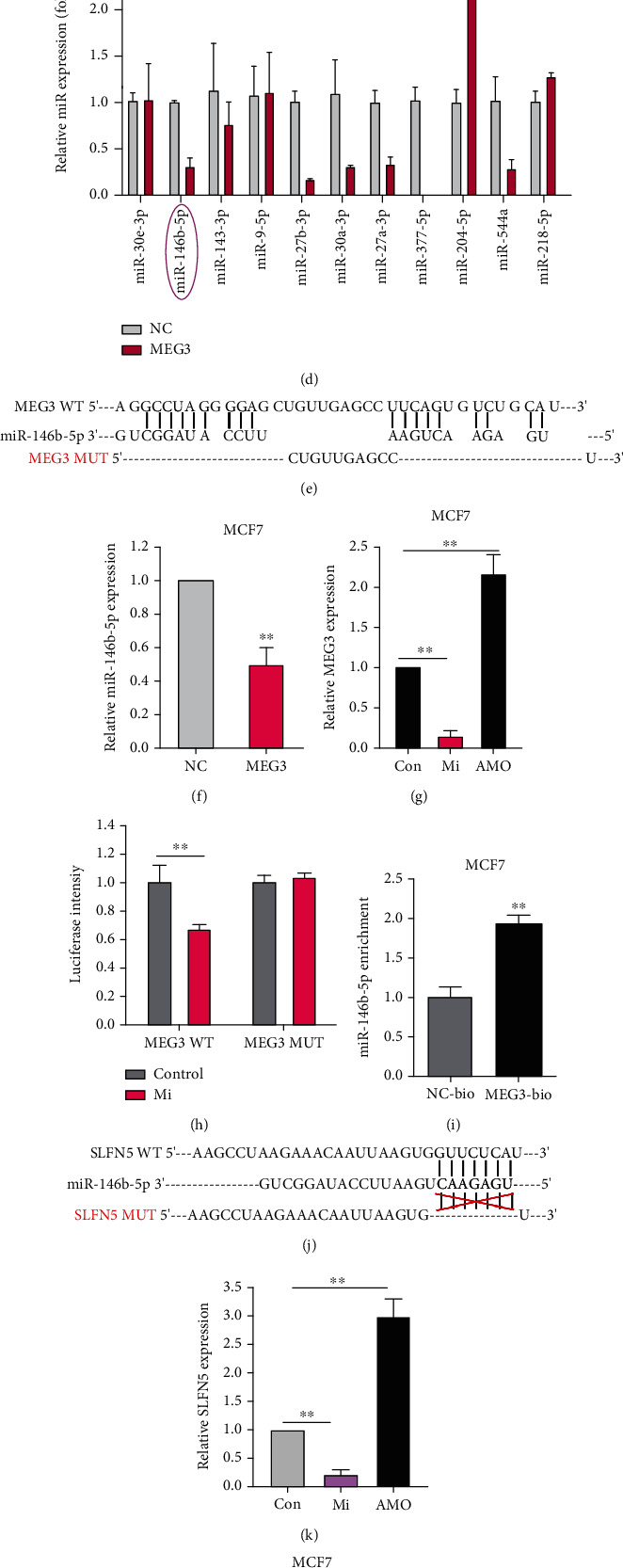
MiR-146b-5p could bind to MEG3 and SLFN5 3′UTR directly by competing endogenous RNAs mechanism. (a, b) Twelve miRNAs predicted to regulate both SLFN5 and MEG3 by bioinformatics. (c, d) Comparison of 12 miRNA expression between control group and si-MEG3/MEG3 group in BRCA cell lines. (e) Schematic representation of binding sites between MEG3 and miR-146b-5p predicted by RNAhybrid. WT: wild type. MUT: mutant. (f) miR-146b-5p expression change in MCF7 cells after MEG3 plasmid transfection. (g) MEG3 expression change after transfection of either mimic (Mi) or inhibitor (AMO) of miR-146b-5p. (h) Luciferase reporter assay in 293T cells after transfection with MEG3 plasmids, either wild type (WT) or mutant (MUT), and Mi of miR-146b-5p. (i) RNA pull down assay of MEG3 binding to miR-146b-5p in MCF7 cells. (j) Schematic representation of binding sites between miR-146b-5p and SLFN5's 3′-UTR predicted by RNAhybrid. (k) Relative mRNA levels of SLFN5 responding to transfection of either mimic (Mi) or inhibitor (AMO) of miR-146b-5p. (l, m) Relative change of SLFN5 protein and mRNA responding to transfection of si-MEG3 or miR-146b-5p Mi or si-MEG3 plus miR-146b-5p inhibitor. (n) Luciferase reporter assay of transfection with SLFN5 plasmid, either WT or MUT, and miR-146b-5p Mi, or si-MEG3, or si-MEG3 plus miR-146b-5p AMO together.

To further explore whether MEG3 regulates SLFN5 RNA expression via a ceRNA mechanism, a hypergeometric test identified 12 miRNAs that could regulate both SLFN5 and MEG3 in the overlapping area between 31 SLFN5-miRNA pairs downloaded from starBase v2.0 and 266 MEG3-miRNA pairs obtained from LncBase v2 (*P* = 0.0122; Figure 4(a)). The network displayed in Figure 4(b) reflects the regulatory relationship among SLFN5, MEG3, and miRNAs. Then, the expressions of 12 miRNAs were analyzed in BRCA cell lines with the loss and gain of MEG3, and miR-146b-5p changed significantly in si-MEG3/MEG3 transfection cells (Figures 4(c) and 4(d)), suggesting that MEG3 can negatively regulate miR-146b-5p expression.

Subsequently, a sequence-based comparison between MEG3 and miR-146b-5p using RNAhybrid revealed that the MEG3 contains a target site for miR-146b-5p (Figure 4(e)). MEG3 overexpression obviously decreased miR-146b-5p level in MCF7 cells (Figure 4(f)). Similarly, MEG3 levels were greatly suppressed by miR-146b-5p mimic (Mi), while evidently elevated by miR-146b-5p inhibitor (AMO) (Figure 4(g)). Further, luciferase activity was detected, and the results showed that miR-146b-5p Mi suppressed the luciferase activity of MEG3-WT in MCF7 cells, but not the mutant one (Figure 4(h)). Then, RNA pull down assay revealed that a higher level of miR-146b-5p was pulled down by biotin-labeled MEG3 (MEG3-Bio) than by NC-Bio (Figure 4(i)), providing evidence for MEG3 binding with miR-146b-5p.

Undoubtedly, a probable binding sequence was found in SLFN5 3′UTR and miR-146b-5p (Figure 4(j)). SLFN5 expression was downregulated by the miR-146b-5p Mi, but was upregulated by AMO (Figure 4(k)). Additionally, treatment with both MEG3 siRNA and miR-146b-5p Mi can decrease SLFN5 protein and mRNA expression (Figures 4(l) and 4(m)). Moreover, miR-146b-5p AMO can restore the response of MEG3 knockdown in T47D and MCF7 cells. Consistently, both miR-146b-5p Mi and MEG3 silence can significantly decrease the luciferase activity of wild-type SLFN5, while did not show response in the mutant group (Figure 4(n)). Moreover, miR-146b-5p inhibitor can restore the response of MEG3 knockdown (Figure 4(n)), suggesting that in BRCA cells MEG3 can promote SLFN5 expression via sponging the miR-146b-5p.

## 4. Discussion

The biological functions of lncRNAs in cancer include cell cycle regulation, microRNA regulation, epigenetic regulation, and signal transduction pathways [31–33]. For example, lncRNA HOTAIR is involved in some cancer malignant progression [34]. However, lncRNA GAS5 played an inhibiting role in renal cell carcinoma [35]. Several studies reported that MEG3 can inhibit cancer cell proliferation in diverse cancers [20, 36, 37]. For example, Li J et al. confirmed that MEG3 inhibits cell growth of cholangiocarcinoma through regulating Bmi/RNF2 [37].

This study analyzed the MEG3 expression in pan-cancers by bioinformatics analysis, and the results demonstrated that MEG3 was decreased in most of cancers. And the downregulation of MEG3 in BRCA is consistent with the previous Dong's research [21]. It brought to our attention that the expression of MEG3 was positively related to SLFN5 by bioinformatics analysis in BRCA patients. Our previous studies revealed that SLFN5 is a tumor suppressor in BRCA [28–30]. SLFN5 can suppress BRCA cell invasion through the downregulation of MT1-MMP [28] and inhibit BRCA cell EMT through the downregulation of ZEB1 [29]. In this study, knockdown of MEG3 can decrease SLFN5 expression, while elevated MEG3 can increase SLFN5 expression in BRCA cells; moreover, loss- and gain-of-function studies showed that MEG3 can inhibit the EMT of BRCA cells similar to SLFN5's function. Furthermore, the overexpression of MEG3 partially reversed morphological/functional changes induced by knockdown of SLFN5. Subsequently, EMT markers (E-cadherin, vimentin, and ZEB1) were analyzed to validate MEG3's roles in keeping epithelial morphology in BRCA cells, which indicates that MEG3 exerts inhibitory functions on BRCA progression by regulating SLFN5. Some studies validated our results; for example, MEG3 may be involved in regulating EMT process to inhibit breast cancer and pituitary development [23, 36]. We also found previously that SLFN5 can inhibit BRCA cell proliferation and promote their apoptosis [30] and that presumably MEG3 may involve in these regulation.

**Figure 5:**
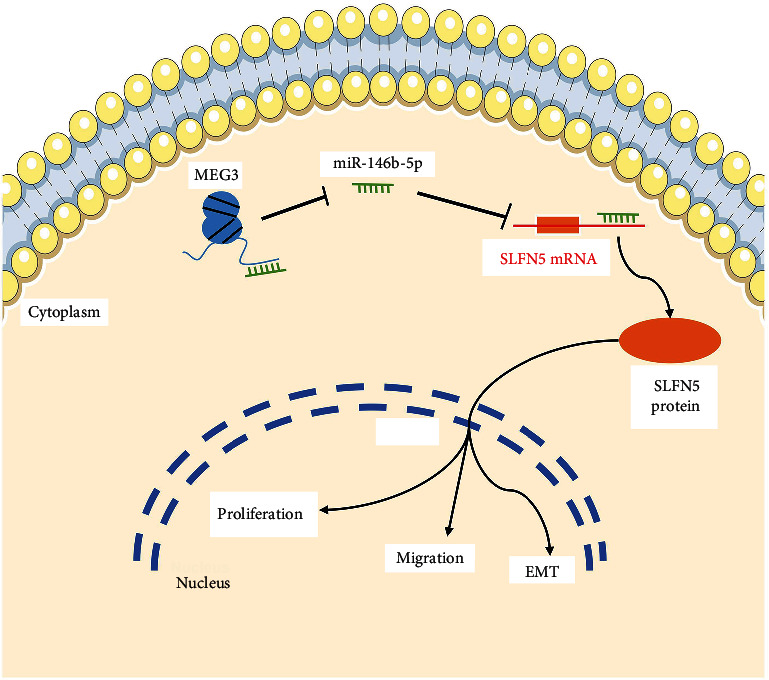
Schematic of MEG3, SLFN5, miR-146b-5p, and the EMT regulatory network in breast cancer cells.

To determine the mechanisms underlying the relationship of MEG3, SLFN5, and EMT, we speculated that the ceRNA network might be a potential regulatory mechanism of MEG3-miRNA-SLFN5 axis. LncRNAs can serve as ceRNAs to sponge miRNAs to regulate the gene expression targeted by miRNAs in diverse cancers [23, 38–40]. Here, our study obtained 12 miRNAs related to both MEG3 and SLFN5 through bioinformatics screening, and the binding sites between miR-146b-5p and MEG3/SLFN5 were found. Subsequently, it is predicted that miR-146b-5p regulates both SLFN5 and MEG3 by RNA hybridization and confirmed that MEG3 can directly bind to miR-146b-5p by RNA pull down assay. However, miR-146b-5p showed the dual functions of promoting or inhibiting tumorigenesis in cancer [41–45]. For instance, Qu L et al. reported that miR-146b-5p can promote Ewing's sarcoma malignancy by inhibiting B-cell translocation gene 2 expression [45]. Notably, the role of miR-146b-5p regulating EMT is controversial in BRCA. Li S et al. reported that lncRNA NEAT1 promotes the BRCA progression by binding miR-146b-5p [46], completely contrary to the results of this study. Meanwhile, Akkiprik M et al. reported that miR-146b-5p is upregulated in the peripheral blood of patients with locally advanced BRCA [47], consistent with the results of this study, but the function of miR-146b-5p in cancers needs be more explored in the near future. Thus, this study confirmed that MEG3 could serve as a ceRNA involved in the potential mechanism to positively modulate SLFN5 expression through miR-146b-5p, thereby affecting EMT and invasion capability in BRCA cells (Figure 5).

## 5. Conclusion

Our present research discovered that MEG3 is weakly expressed in BRCA and regulates SLFN5 expression positively *in vitro*. Importantly, MEG3 modulates SLFN5 expression via sponging miR-146b-5p and inhibits EMT of BRCA cells, which indicates that MEG3/miR-146b-5p/SLFN5 axis may be a potential therapeutic target in BRCA treatment.

## Data Availability

The data used to support the findings of this study are included within the article.

## Supplementary Materials

Supplementary 1 Figure S1: Comparison of clinical characteristics in patients with breast cancer.

Supplementary 2 Table 1: Association of MEG3 expression with clinical characteristics in breast cancer. Table 2: The siRNA, microRNA mimic and inhibitor sequences in this study. Table 3: The primer sequences of all RNAs in this study.
