# Supplementary figures and images for: The Long Noncoding RNA MEG3 Retains Epithelial-Mesenchymal Transition by Sponging miR-146b-5p to Regulate SLFN5 Expression in Breast Cancer Cells
Source: J Immunol Res. 2022 Aug 18;2022:1824166. doi: 10.1155/2022/1824166 (PMC9411926; doi:10.1155/2022/1824166)

Figure S1

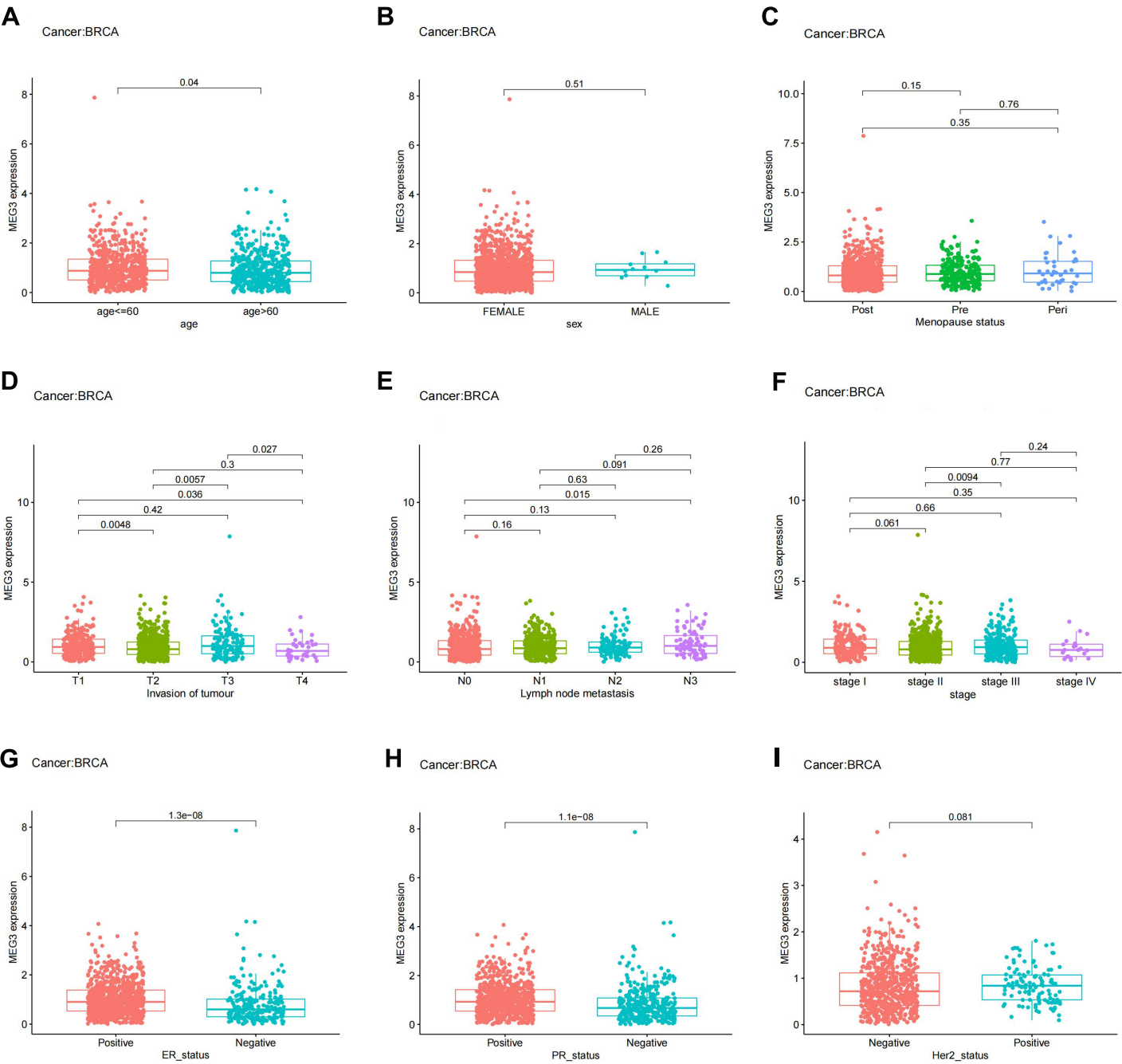

Supplement: Supplementary 1 — Figure S1: Comparison of clinical characteristics in patients with breast cancer. [file 1824166.f1.pdf]
