# Supplementary material for: The Long Noncoding RNA MEG3 Retains Epithelial-Mesenchymal Transition by Sponging miR-146b-5p to Regulate SLFN5 Expression in Breast Cancer Cells
Source: J Immunol Res. 2022 Aug 18;2022:1824166. doi: 10.1155/2022/1824166 (PMC9411926; doi:10.1155/2022/1824166)
Supplement: Supplementary 2 — Table 1: Association of MEG3 expression with clinical characteristics in breast cancer. Table 2: The siRNA, microRNA mimic and inhibitor sequences in this study. Table 3: The primer sequences of all RNAs in this study. [file 1824166.f2.zip › supple Table 1 (1).DOCX]

**Table 1** The clinical characteristics associated with MEG3 expression in breast cancer

| Variables | Number | Mean (MEG3) | P value^#^ | | |
| --- | --- | --- | --- | --- | --- |
| Age (years) |  |  |  |  |  |
| >60 | 489 | 0.93±0.68 |  |  |  |
| ≤60 | 607 | 1.01±0.72 | 0.04 |  |  |
| Sex |  |  |  |  |  |
| Male | 12 | 0.98±0.40 |  |  |  |
| Female | 1085 | 0.98±0.71 | 0.51 |  |  |
| Menopause status |  |  |  |  |  |
| Peri | 40 | 1.10±0.81 |  |  |  |
| Post | 703 | 0.96±0.73 | 0.35(vs. Peri) |  |  |
| Pre | 229 | 0.99±0.60 | 0.15 | 0.76 (vs. Post) |  |
| Invasion of tumour |  |  |  |  |  |
| T1 | 281 | 1.04±0.69 |  |  |  |
| T2 | 641 | 0.92±0.65 | 0.0048 (vs. T1) |  |  |
| T3 | 138 | 1.19±1.01 | 0.0057 | 0.42 (vs. T2) |  |
| T4 | 40 | 0.82±0.61 | 0.027 | 0.3 | 0.036 (vs. T3) |
| Lymph node metastasis |  |  |  |  |  |
| N0 | 518 | 0.96±0.78 |  |  |  |
| N1 | 367 | 0.98±0.65 | 0.16 (vs. N0) |  |  |
| N2 | 120 | 1.00±0.62 | 0.13 | 0.63(vs. N1) |  |
| N3 | 78 | 1.18±0.80 | 0.015 | 0.091 | 0.26(vs. N2) |
| pTNM-Stage |  |  |  |  |  |
| Stage I | 182 | 1.04±0.73 |  |  |  |
| Stage II | 628 | 0.94±0.72 | 0.061 (vs. Stage I) |  |  |
| Stage III | 250 | 1.06±0.72 | 0.66 | 0.0094 (vs. Stage II) |  |
| Stage IV | 20 | 0.88±0.63 | 0.35 | 0.77 | 0.24 (vs. Stage III) |
| ER_status |  |  |  |  |  |
| Negative | 238 | 0.83±0.83 |  |  |  |
| Positive | 809 | 1.02±0.67 | 1.30E-08 |  |  |
| PR_status |  |  |  |  |  |
| Negative | 344 | 0.85±0.76 |  |  |  |
| Positive | 700 | 1.05±0.68 | 1.10E-08 |  |  |
| Her2_status |  |  |  |  |  |
| Negative | 647 | 0.82±0.56 |  |  |  |
| Positive | 114 | 0.85±0.38 | 0.081 |  |  |

p value was calculated by a student’s t-test; #, refer to Figure 1B; Abbr. ER, estrogen receptor; PR, progesterone receptor.
