# Supplementary material for: The Long Noncoding RNA MEG3 Retains Epithelial-Mesenchymal Transition by Sponging miR-146b-5p to Regulate SLFN5 Expression in Breast Cancer Cells
Source: J Immunol Res. 2022 Aug 18;2022:1824166. doi: 10.1155/2022/1824166 (PMC9411926; doi:10.1155/2022/1824166)
Supplement: Supplementary 2 — Table 1: Association of MEG3 expression with clinical characteristics in breast cancer. Table 2: The siRNA, microRNA mimic and inhibitor sequences in this study. Table 3: The primer sequences of all RNAs in this study. [file 1824166.f2.zip › supple Table 2 (1).DOCX]

**Table 2** siRNA, microRNA mimic and inhibitor sequence

| Sequence name | Sequence |
| --- | --- |
| MEG3 siRNA-1 | 5′-GCUCAUACUUUGACUCUAUTT-3′ |
| MEG3 siRNA-2 | 5′-GGAUCCCACCAACAUACAATT-3′ |
| SLFN5 siRNA | 5′-GACUCAGACUCCAACGAAUTT-3′ |
| hsa-miR-146b-5p mimic | 5'-UGAGAACUGAAUUCCAUAGGCUG-3' |
| hsa-miR-146b-5p inhibitor | 5'-CAGCCUAUGGAAUUCAGUUCUCA-3' |
