# Supplementary material for: The Long Noncoding RNA MEG3 Retains Epithelial-Mesenchymal Transition by Sponging miR-146b-5p to Regulate SLFN5 Expression in Breast Cancer Cells
Source: J Immunol Res. 2022 Aug 18;2022:1824166. doi: 10.1155/2022/1824166 (PMC9411926; doi:10.1155/2022/1824166)
Supplement: Supplementary 2 — Table 1: Association of MEG3 expression with clinical characteristics in breast cancer. Table 2: The siRNA, microRNA mimic and inhibitor sequences in this study. Table 3: The primer sequences of all RNAs in this study. [file 1824166.f2.zip › supple Table 3 (1).DOCX]

**Table 3** Primer sequence

| **Homo gene name** | **forward (F) and reverse (R) primer** |
| --- | --- |
| **hsa-miR-146b-5p** | F: 5’-ACACTCCAGCTGGGTGAGAACTGAATTCCATAG-3’  R: 5’ -CTCAACTGGTGTCGTGGA-3’ |
| **MEG3**  **(NR_002766.2)** | F: ATCATCCGTCCACCTCCTTGTCTTC-3’  R: GTATGAGCATAGCAAAGGTCAGGGC-3’ |
| **SLFN5**  **(NM_44975.3)** | F: 5′-CATCCGACGCATCACCGATCTG-3′  R: 5′-CATCCGACGCATCACCGATCTG-3′ |
| **E-cadherin (NM_004360.4)** | F: 5'-CTGAGAACGAGGCTAACG-3'  R: 5'-GTCCACCATCATCATTCAATAT-3' |
| **vimentin**  **(NM_003380.3)** | F: 5'-TCGTGAATACCAAGACCTGCTCAATG-3'  R: 5'-AATCCTGCTCTCCTCGCCTTCC-3' |
| **ZEB1**  **(NM_001128128.2)** | F: 5'-TGGCGGTAGATGGTAATGTAATAAGGC-3'  R: 5'-GCTAGGCTGCTCAAGACTGTAGTTG-3' |
